# Selective deletion of FGFR1 in AgRP neurons impairs energy homeostasis under high-fat diet in mice

**DOI:** 10.1016/j.molmet.2026.102332

**Published:** 2026-02-10

**Authors:** Daniel Shookster, Shea O'Connell, Patel Darshan, Taylor Landry, Wyatt Bunner, Zhiying Jiang, Qingchun Tong, Hu Huang

**Affiliations:** 1 East Carolina Diabetes and Obesity Institute, East Carolina University, Greenville, NC, USA; 2 Department of Kinesiology, East Carolina University, Greenville, NC, USA; 3 Human Performance Laboratory, College of Human Performance and Health, East Carolina University, Greenville, NC, USA; 4 Brown Foundation Institute of Molecular Medicine of McGovern Medical School, University of Texas Health Science Center at Houston, Houston, TX, USA; 5 Department of Physiology, East Carolina University, Greenville, NC, USA

**Keywords:** FGFR1, AgRP neuron, Energy homeostasis, High fat diet

## Abstract

**Background:**

The global obesity crisis and the limited success of current treatments underscore the need to identify novel regulatory pathways. While central administration of α-Klotho exerts anti-obesity effects in rodents through AgRP neurons, the intracellular signaling mechanisms that mediate this process remain undefined.

**Methods:**

To define the role of FGFR1 within the α-Klotho signaling pathway in AgRP neurons, we performed a targeted deletion of the receptor in adult mice using an AAV-mediated CRISPR/Cas9 system alongside transgenic models.

**Results:**

Deletion of FGFR1 in AgRP neurons disrupted energy homeostasis, promoting weight gain induced by a high-fat diet. Electrophysiological recordings revealed that FGFR1 loss increased the intrinsic firing rate of AgRP neurons and abolished the suppressive effect of α-Klotho on their activity. At the molecular level, FGFR1 knockdown decreased phosphorylation of the transcription factor FOXO1 and elevated AgRP mRNA expression.

**Conclusions:**

Our results define a crucial FGFR1 signaling axis in AgRP neurons that coordinately regulates their electrical activity and peptide expression, thereby establishing FGFR1 as an essential regulator of energy homeostasis.

## Highlights

• Deleting FGFR1 in adult mouse AgRP neurons via AAV CRISPR-Cas9 reveals its critical role in energy balance.
• AgRP neuron FGFR1 deletion disrupts energy balance, causing high-fat diet-induced weight gain.
• FGFR1 is essential for AgRP neuron excitability; its loss increases firing and blocks α-Klotho suppression.
• FGFR1 loss reduces FOXO1 phosphorylation and elevates AgRP expression.
• FGFR1 signaling coordinates AgRP neuron firing and gene expression to control body weight.

## Graphical abstract

Proposed mechanisms by which FGFR1 regulates AgRP neuron activity and FOXO1 phosphorylation in the hypothalamic cell line.

**Figure:**
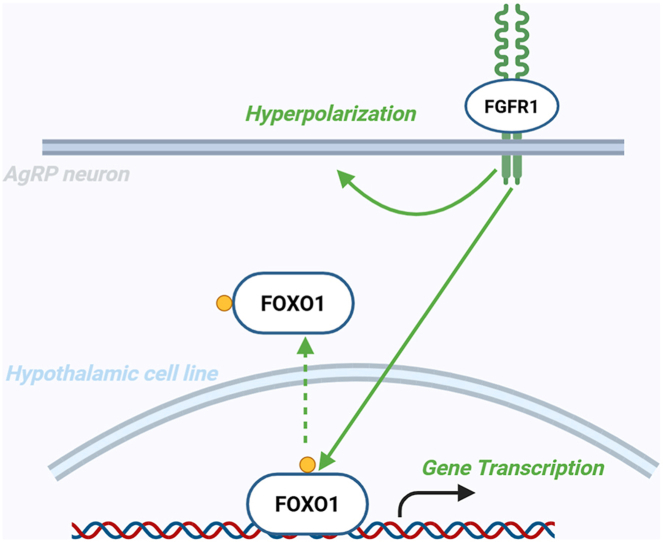

## 1 Introduction

Obesity is the primary risk factor for the development of T2D and other diseases such as hypertension and cardiovascular disease [1]. Obesity is often associated with an energy imbalance that contributes to dysregulation of body-weight homeostasis. The central nervous system (CNS) serves as the principal orchestrator of systemic energy homeostasis, dynamically integrating a suite of peripheral metabolic signals to modulate behavior and physiology [2]. Key CNS regions, including the hypothalamus, express receptors for circulating hormones such as leptin, insulin, and ghrelin, which relay critical information regarding long-term energy reserves and short-term nutritional status. Within hypothalamic nuclei like the arcuate nucleus, specialized neuronal populations—notably, orexigenic agouti-related peptide (AgRP)/neuropeptide Y (NPY) neurons and anorexigenic pro-opiomelanocortin (POMC) neurons—process these afferent signals to regulate food intake and energy expenditure [3,4].

Emerging evidence has positioned hypothalamic signaling through the fibroblast growth factor receptor 1 (FGFR1) as a pivotal regulator of energy homeostasis. This receptor serves as a common target for a suite of metabolic hormones, including FGF21, FGF19, and FGF4, which exert potent anti-obesity and anti-diabetic effects through actions in the central nervous system [[5], [6], [7], [8], [9], [10], [11], [12], [13], [14], [15], [16]]. Our laboratory contributed to this paradigm by demonstrating that α-klotho, a circulating factor that declines in human obesity, functions as a novel humoral agent within the hypothalamus. We found that central administration of α-klotho elicits robust anti-diabetic and anorexigenic effects in rodent models by specifically inhibiting FGFR1 on AgRP neurons. Crucially, the therapeutic benefits of α-klotho are entirely dependent on FGFR1 activation, as its pharmacological inhibition abolishes both the neuronal responses and the metabolic improvements [[17], [18], [19]].

Despite the clear implication of FGFR1 as a mediator for multiple metabolic hormones, its precise and necessary role within defined hypothalamic neuronal circuits has not been comprehensively addressed. A major limitation has been the methodological challenge of isolating its function in adulthood. Conventional germline or developmental knockout strategies are confounded by potential compensatory mechanisms [20], such as the redundancy of FGF ligands or the rewiring of neural circuits during development, which can mask the true physiological function of the receptor in the mature animal. Consequently, the essential cell-type-specific role of FGFR1 in regulating energy balance in the adult hypothalamus remains a fundamental and unresolved question.

To directly address this gap, we developed a targeted strategy to manipulate FGFR1 expression specifically in mature hypothalamic neurons, thereby eliminating the confounds of developmental compensation. We employed an Adeno-Associated Virus (AAV)-mediated Cre-dependent CRISPR-Cas9 system to achieve selective loss-of-function of FGFR1 in adult mice. This approach allowed us to probe the metabolic necessity and sufficiency of FGFR1 within discrete neuronal populations post-developmentally. Using this model, we demonstrate that the deletion of FGFR1 specifically in ARC AgRP neurons is sufficient to drive increased weight gain, adiposity, and glucose intolerance, phenotypes that are markedly induced under the challenge of a high-fat diet. These findings identify FGFR1 signaling within AgRP neurons as a critical regulator of systemic energy homeostasis, offering a promising novel target for therapeutic intervention in metabolic disease.

## 2 Methods

### 2.1 Mice

*AgRP-IRES-Cre* mice (JAX 012899) mice were housed at 20–22 °C with a 12-h light–dark cycle (7:30 AM Lights on/7:30 PM Lights off). Mice were fed a high-fat diet (Research diet D12331) with ad libitum access to water throughout the study. All experimental protocols were approved by the Institutional Animal Care and Use Committees of East Carolina University, and mice were cared for in accordance with the National Institutes of Health Guide for the Care and Use of Laboratory Animals. Both male and female mice were included in the study and statistically analyzed for sex-specific effects; no significant differences were found.

### 2.2 Stereotaxic injections and viral constructs

Six-week-old mice were anesthetized with 2–4% isoflurane and then placed into a stereotaxic device (KOPF model 922), after which eye ointment was applied, and a small incision was made to expose the skull. A cordless micro-drill (Stoelting, Wood Dale, IL) was then used to drill a small hole in the skull to allow for needle passage into the brain. Bilateral injections of viral constructs (250 nl/side) were targeted to the ARC according to coordinates from bregma, anterior-posterior (AP): −1.40 mm, dorsal-ventral (DV): −5.85 mm, left-right (LR): ±0.3 mm at a rate of 30 nl/min using a 0.5 μl Hamilton syringe (Neuros model 7000.5 KH). Following injections, the needle was left in position for an additional 10 min to permit virus diffusion prior to withdrawing the needle. All viral constructs were sourced from Vector Builder Inc. (Chicago, IL, USA) and stored at −80 °C until use. Based on the manufacturer's description and online CRISPR tool (http://crispr.mit.edu/), the sgRNAs to detect FGFR1 are located at exon 5 (CGGTGAAGTTCAAGTGCCCGTCGAGT) and exon 10 (TCTCCGAATATGAGCTCCCTGAGGAT), respectively, and a scramble sgRNA (GTGTAGTTCGACCATTCGTG-GGGAGT) was used for control. Using 2 sgRNAs to ensure the specificity and avoid off-target for FGFR1. Mice with total misses or partial expression were excluded from analysis after examining mCherry expression. For postoperative care, mice were injected intraperitoneally with meloxicam (0.5 mg/kg BW) for three consecutive days, with their body weight and health conditions being closely monitored during recovery.

### 2.3 Food intake, body weight, body composition, and energy expenditure

Following AAV injections, food intake and body weight were measured weekly during the two-week recovery period and daily thereafter. Food intake was measured daily between 8:00 and 9:00 AM at the age of 8–10 weeks old using an OHAUS scale (VALOR™ 2000, OHAUS, Parsippany, NJ, USA). During measurements, cages were inspected for food pellets and partially eaten food bits to ensure accurate measurements. For energy expenditure measurements, 12-week-old mice were singly housed in TSE PhenoMaster metabolic cages (TSE PhenoMaster, Berlin, Germany). Mice were acclimatized for three days before recording, with data collected during days 4–6 used for analysis. Energy expenditure data were analyzed using the online CalR tool [21,22]. At the end of the study, body composition was determined using Echo MRI (Echo MRI 3-in-1 composition analyzer, Echo Medical Systems, Houston, TX).

### 2.4 Intracerebroventricular cannulation (ICV)

Following AAV microinjections at six weeks of age, mice were allowed to recover for two weeks before ICV cannulation. ICV cannulations were performed as previously described [17]. In brief, mice were anesthetized with 4% isoflurane and then placed into a stereotaxic device (KOPF model 922) with ear bars. A cordless micro-drill (Stoelting, Wood Dale, IL) was then used to drill a small hole in the skull (1.0 mm lateral, −0.5 mm posterior, 2.5 mm deep to the bregma), and a cannula was placed into the lateral ventricle and secured with 3M carboxylate dental cement. For postoperative care, mice were injected intraperitoneally with meloxicam (0.5 mg/kg) for three consecutive days and recovered for two weeks before experiments. Two weeks after cannulation (10 weeks old), mice were fasted overnight, and the next morning, 2.0 μg recombinant α-klotho (R&D Systems) or saline was administered via a Hamilton syringe at a volume of 2.0 μL. Ninety minutes after administration, mice were intracardially perfused with PBS followed by 10% formalin (Epredia™ Formal-Fixx™). Brains were then incubated at 4 °C in 10% formalin for 48 h before being stored in 30% sucrose until sectioning. Cannula placements were verified during sectioning, and mice with misguided/misplaced cannulas were excluded from analysis.

### 2.5 Glucose and insulin tolerance tests

At 14 weeks of age (8 weeks post-AAV), mice underwent glucose tolerance tests by intraperitoneal injections using a 20% glucose solution (1.0 g/kg BW) after an overnight fast. Insulin tolerance tests were performed at 16 weeks of age (10 weeks post-AAV) by intraperitoneal injection of insulin 0.6U/kg after a 4-hour fast. Blood glucose levels were measured from a drop of tail blood at 0, 15, 30, 60, 90, and 120-minute time points during the tests using a handheld glucometer (ReliOn Prime Blood Glucose Monitoring System; ARKRAY Inc., Kyoto, Japan).

### 2.6 Hematoxylin and eosin (H&E) staining

Tissues were fixed overnight in 4% PFA at 4 °C, then washed in PBS before being embedded in cutting medium (Richard-Allan Scientific Neg-50 Frozen Section Medium) and cut into 10 μm sections using a cryostat (Microm HM 550). Sections were then stained with Mayer's hematoxylin solution (Sigma–Aldrich, MHS80) and Eosin Y (Sigma–Aldrich, 318906), after which images were captured on a Keyence BZ-X810 digital microscope (Keyence, Itasca, IL, USA) using 10x and 20× objective lenses.

### 2.7 Oil red O staining

Liver tissues were fixed overnight in 4% PFA at 4 °C, then washed in PBS before being embedded in cutting medium (Richard-Allan Scientific Neg-50 Frozen Section Medium) and cut into 10 μm sections using a cryostat (Microm HM 550). Once cut, the sections were stained with oil red o (Sigma–Aldrich, 1.02419), then counterstained with hematoxylin (Sigma–Aldrich). Images were captured on a Keyence BZ-X810 microscope (Keyence, Itasca, IL, USA) using 10x and 20× objective lenses. Lipid content was quantified using Fiji software [23] as previously described [24].

### 2.8 Quantitative real-time PCR

RNA was isolated from tissues or cells using the RNeasy Micro Kit (Qiagen, 74004) according to the manufacturer's protocol. Following isolation, RNA was reverse transcribed into cDNA using the Applied Biosystems high-capacity cDNA reverse transcription kit (Thermo Fisher, 4368814) according to the manufacturer's protocol. RT-qPCR was then performed in triplicate using the Applied Biosystems ViiA 7 system, with mRNA expression normalized to the mouse 18s ribosomal RNA housekeeping gene and expression fold change calculated using the 2^−^ΔΔCt method. Primer sequences are listed in Supplemental Table 1.

### 2.9 Western blot

Western blot procedures were performed as previously described [17]. In brief, following the extraction of protein according to standard protocol, the Pierce BCA Protein Assay Kit was used to assess protein concentration. Electrophoresis was conducted on a 4–20% Criterion™ Tris–HCl Protein Gel loaded, then transferred to a nitrocellulose membrane and blocked with 5% non-fat milk TBS-T for 2 h. Following the blocking period, primary antibodies pFOXO1^ser256^, total FOXO1, or FGFR1 (Cell Signaling Technology) were applied 1:1000, after which the membranes were incubated at 4 °C overnight. The following day, secondary antibodies IgG-HRP 1:1000 (Santa Cruz Biotechnology) were applied, followed by incubation at room temperature for 2 h. Membranes were washed with TBS-T between each procedure, and immunoreactive bands were visualized using ECL methods and imaged with Bio-Rad ChemiDoc.

### 2.10 Cell culture and transfection

For the knockdown study, hypothalamic GT1-7 cells were used to test viral constructs. Hypothalamic GT1-7 cells were cultured at 37**°**C, 5% CO_2_ in high-glucose (4.5 mg/dL) DMEM, 10% FBS, and 1% penicillin-streptomycin with adenovirus Cre to permit the recombination of Cre-dependent constructs. Cells were then transfected with lipofectamine 3000 (ThermoFisher Scientific) according to the manufacturer's protocol using DNA plasmids of pAAV-sagRNA^FGFR1^-FLEX-mCherry or pAAV-sagRNA^Scramble^-FLEX-mCherry and pAAV-FLEX-SaCas9 for knockdown experiments. Briefly, cells were incubated for 72 h following transfection, after which cells were treated with 3.65 mM α-Klotho (R&D Systems) or vehicle control for 30 min, followed by protein extraction and electrophoresis to investigate intracellular signaling pathways.

### 2.11 Immunohistochemistry

Mice were intracardially perfused with PBS followed by 10% formalin (Epredia™ Formal-Fixx™), after which brains were incubated at 4 °C in 10% formalin for 48 h before being stored in 30% sucrose until sectioning. Brains were sectioned into 30 μm coronal sections using a freezing microtome (VT1000 S; Leica, Wetzlar, Germany). After sectioning, brain slices were washed in PBS (6 × 10 min), then blocked for 1 h in PBST & normal donkey serum (Jackson ImmunoResearch Inc., West Grove, PA, USA), then incubated overnight with primary antibodies for cFOS 1:500 (Santa Cruz Biotechnology). The following day, sections were washed in PBS (6 × 10min) and then incubated with Alexa-flourophore secondary antibodies (Abcam, Waltham Boston, MA, USA) for 1 h. Sections were then mounted on SuperFrost slides (Fisher Scientific) with VECTASHIELD® Antifade Mounting Medium with DAPI H-1200-10 (Vector Laboratories Inc., Burlingame, CA, USA). Images were taken on a Keyence BZ-X810 microscope (Keyence, Itasca, IL, USA) using 10x and 20× objective lenses and analyzed using Fiji software.

### 2.12 Fluorescence activated cell sorting (FACS)

Following AAV injection and euthanasia, the arcuate nucleus (ARC) was dissected from the hypothalamus of 9–10 week-old adult mice, then enzymatically digested using Papain dissociation (Worthington Papain) as previously described [25]. Following dissociation, Dapi was added to the suspension to aid in cell sorting to ensure cell health, after which, cells were sorted using a BD FACS Aria Fusion device (BD Bioscience). RNA was extracted for quantitative real-time PCR analysis from either mCherry-positive or mCherry-negative cells exclusively. Cell sorting was conducted using measurements of mCherry and Dapi fluorescence (mCherry: excitation 561 nm, detection: bandpass 610 ± 20 nm, Dapi: excitation 405 nm, detection: bandpass 450 ± 50 nm).

### 2.13 Electrophysiology

Electrophysiological experiments were conducted in acutely prepared hypothalamic slices, as previously described [17]. Briefly, 8- to 10-week-old adult mice were deeply anesthetized with a mixture of ketamine/xylazine (intraperitoneally) and transcardially perfused with ice-cold cutting solution containing the following (in mM): 75 sucrose, 73 NaCl, 26 NaHCO3, 2.5 KCl, 1.25 NaH2PO4, 15 glucose, 7 MgCl2, and 0.5 CaCl2, saturated with 95% O2/5% CO2. The brains were quickly removed and blocked, with the rostral face of the block glued to the specimen plate of the buffer tray then immersed in an ice-cold cutting solution. Coronal slices (280 μm) containing ARC were sectioned using a Leica VT1000S Vibratome and transferred to a holding chamber with artificial CSF (aCSF) containing the following (in mM): 123 NaCl, 26 NaHCO3, 2.5 KCl, 1.25 NaH2PO4, 10 glucose, 1.3 MgCl2, and 2.5 CaCl2 and saturated with 95% O2/5% CO2 at 31–33 °C for 30 min, then maintained at room temperature for at least 1 h to allow for recovery before any electrophysiological recordings. Equivalent length periods (0.5–5 min) were set within each recording during perfusion of aCSF or α-klotho (3.65 mmol/L).

### 2.14 Statistical analyses

Data are presented as mean ± SEM, and relevant statistical test information and results are presented in the figure legends. Analyses were made using GraphPad Prism statistic software versions 8 & 9 (GraphPad Software, La Jolla, CA) with a P value < 0.05 considered statistically significant.

## 3 Results

### 3.1 Viral Strategy and validation of CRISPR/Cas9-mediated deletion of FGFR1 in AgRP neurons

**Figure 1:**
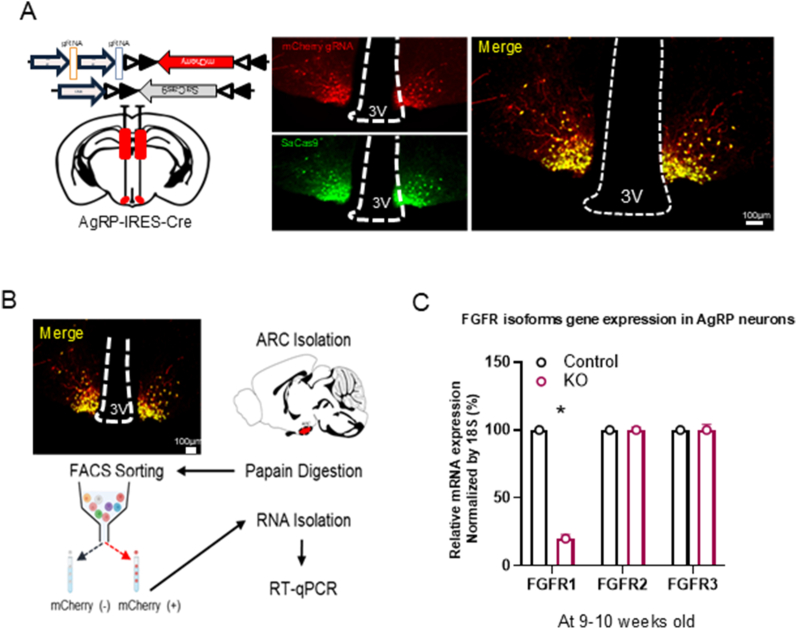
**Viral Strategy and validation of CRISPR/Cas9-mediated deletion of FGFR1 in AgRP neurons**. **A)** Schematic diagram of viral constructs bilaterally injected into the ARC of AgRP-IRES-Cre mice, and Co-immunostaining of mCherry and SaCas9 expression in AgRP neurons at the age of 9–10 weeks old mice. **B)** Schematic Diagram of experimental workflow for FACS sorting of AgRP neurons based on mCherry expression. **C)** FGFR1, 2, and 3 mRNA expression following CRISPR-Cas9-mediated deletion of FGFR1 in AgRP neurons, n = 3 per group (Welch's t test, Two-tailed t = 11.74, df = 2.210, p = 0.0049).

**Supplemental Figure 1:**
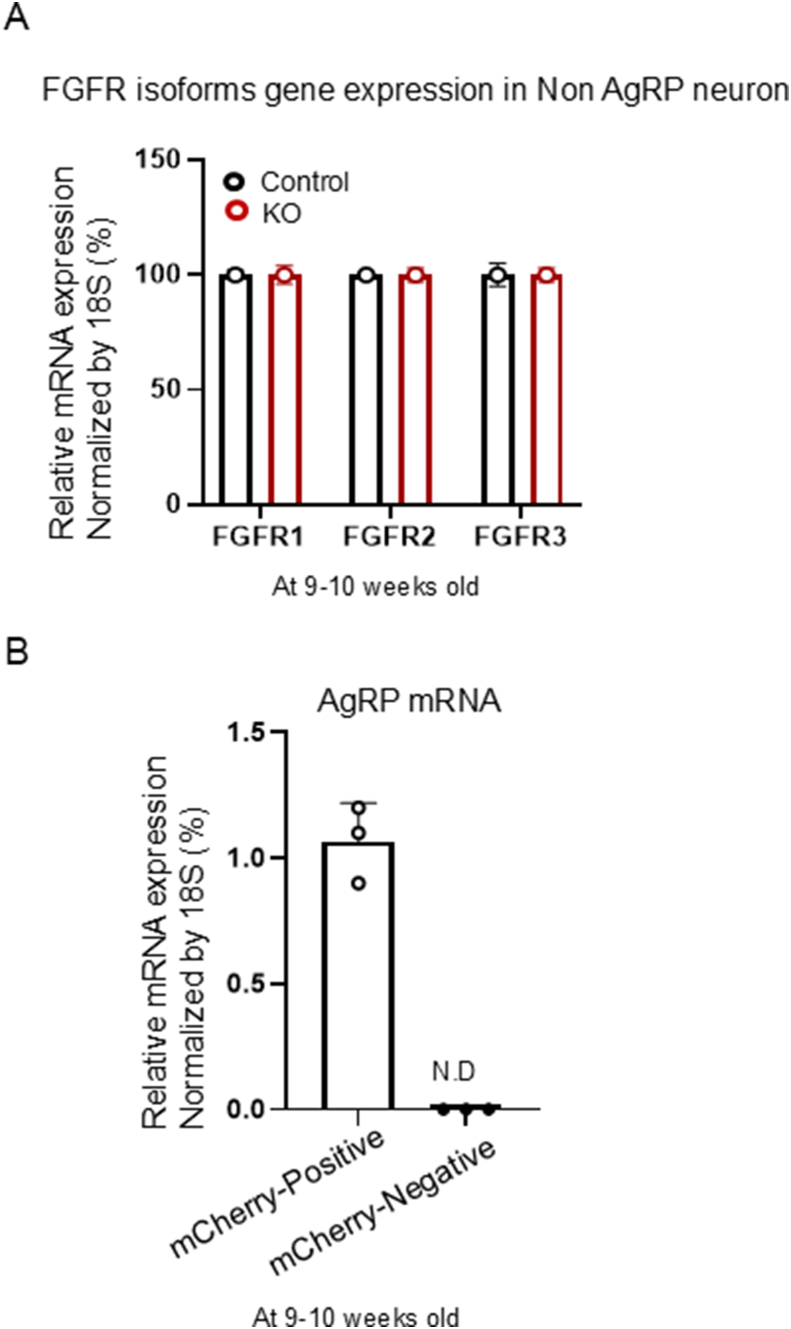
Viral Strategy and validation of CRISPR/Cas9-mediated deletion of FGFR1 in AgRP neurons. After fluorescence-activated cell sorting (FACS), A) FGFR1, 2, and 3 mRNA expression following CRISPR-Cas9-mediated deletion of FGFR1 in non-AgRP (mCherry negative) neurons, n = 3 per group, no significance was observed. B) AgRP mRNA expression in mCherry-Positive and mCherry-Negative neurons following AAV CRISPR-Cas9-mediated deletion of FGFR1, n = 3 per group. All data were collected from mice at the age of 9–10 weeks old.

To investigate the metabolic role of FGFR1 in hypothalamic neurons, we employed targeted loss-of-function approaches using Adeno-Associated Virus (AAV) mediated Cre-dependent CRISPR-Cas9 system (Figure 1A) [[26], [27], [28]], coupled with transgenic mice, to selectively manipulate FGFR1 expression in ARC AgRP neurons of adult mice. Allowing us to eliminate potential compensatory mechanisms during development and off-target effects of chemical inhibition, undesired effects highlighted by work showing that the neural circuits governing energy balance are highly plastic and sensitive to developmental compensations [[29], [30], [31], [32], [33]]. For validating the AAVs injection, we observed that co-expression of mCherry and saCas9 was restricted to the virus-transduced ARC and not present in the adjacent ventromedial hypothalamus, which does not express Cre recombinase in our mouse model (Fig. 1A). Furthermore, to ensure the observed results were not a byproduct of the CRISPR gRNA or SaCas9 protein, control mice were co-injected with a scramble gRNA and SaCas9 designed in the same manner. To assess the efficacy of CRISPR/Cas9-mediated FGFR1 deletion in vivo, we performed fluorescence-activated cell sorting (FACS) to isolate mCherry-positive AgRP neurons and mCherry-negative non-AgRP neurons from the arcuate nucleus (ARC) of AAV-injected mice. RT–qPCR analysis of these populations revealed a marked reduction in FGFR1 mRNA expression in mCherry-positive neurons from mice injected with AAVs encoding FGFR1-targeting sgRNAs, compared to controls receiving a scramble sgRNA. In contrast, expression of other isoforms (FGFR2 and FGFR3) was unchanged (Fig. 1B, C). Furthermore, all three FGFR isoforms remained intact in mCherry-negative non-AgRP neurons, in which AgRP mRNA was undetectable (Supplementary Fig. 1). Together, these results demonstrate effective and specific CRISPR/Cas9-mediated deletion of FGFR1 in AgRP neurons.

### 3.2 High-fat diet feeding induces weight gain, adiposity, and glucose intolerance following CRISPR/Cas9-mediated deletion of FGFR1 in AgRP neurons

**Figure 2:**
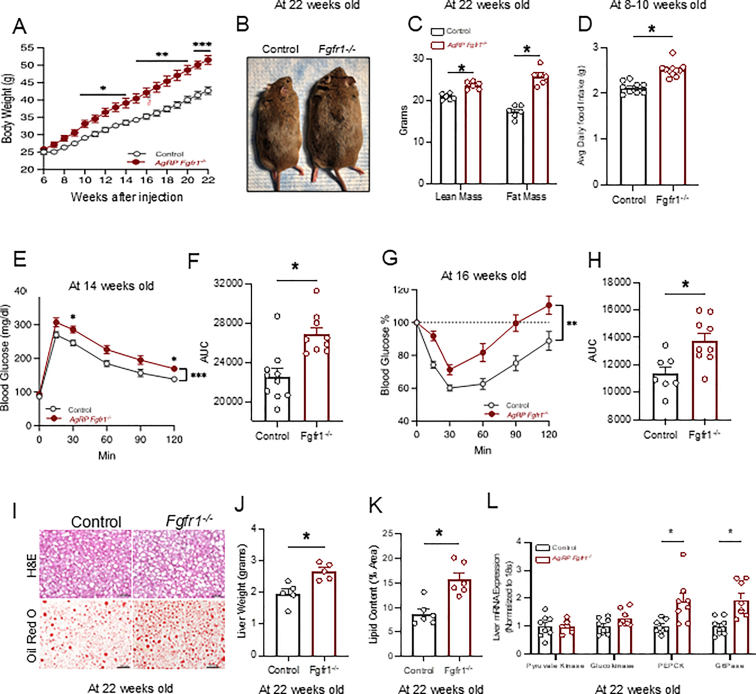
**High-fat diet feeding induces weight gain and adiposity and glucose intolerance following CRISPR/Cas9-mediated deletion of FGFR1 in AgRP neurons**. **A)** Body weight curve after injection (control n = 9, AgRP FGFR1−/− n = 11). Two-way ANOVA (F (1, 18) = 17.52, P = 0.0006). **B)** Representative image of littermates at the age of 22 weeks old, **C)** Body composition analysis was performed at the age of 22 weeks old (n = 6 per group), unpaired students t tests two tailed (lean mass-t = 5.990, df = 9.595, p = 0.000158; fat mass-t = 8.411, df = 9.194, p = 0.000026). **D)** Daily food intake was measured at the age of 8–10 weeks old (control n = 9, AgRP FGFR1−/− n = 11) Welch's two tailed t test, t = 6.292, df = 17.94, p= <0.0001). **E, F)** Glucose tolerance test and AUC were performed at the age of 14 weeks old (control n = 10, AgRP FGFR1−/− n = 9) Two-way ANOVA F (1, 17) = 18.52, P = 0.0005; AUC Welch's two tailed t test t = 4.018, df = 16.75, p = 0.0009). **G, H)** Insulin tolerance test and AUC were performed at the age of 16 weeks old (control n = 8, AgRP FGFR11−/− n = 9) Two-way ANOVA F (1, 14) = 15.31, P = 0.0016; AUC Welch's two tailed t test t t = 3.185, df = 13.97 P = 0.0066). **I)** Liver H&E (top) and Oil Red O stains were performed at the age of 22 weeks old. **J)** Liver weights were measured at the age of 22 weeks old (n = 5 per group) Welch's two tailed t test t = 3.567, df = 7.347, P = 0.0084). **K)** Oil Red O lipid quantification was measured at the age of 22 weeks old (n = 6 per group) Welch's two tailed t test, t = 4.261, df = 9.243. **L)** mRNA expression of gluconeogenic genes from the liver was measured at the age of 22 weeks old (n = 8 per group). Data are presented as mean ± s.e.m. ∗*P* < 0.05, ∗∗*P* < 0.01, ∗∗∗*P* < 0.001, ∗∗∗∗*P* < 0.0001.

**Supplemental Figure 2:**
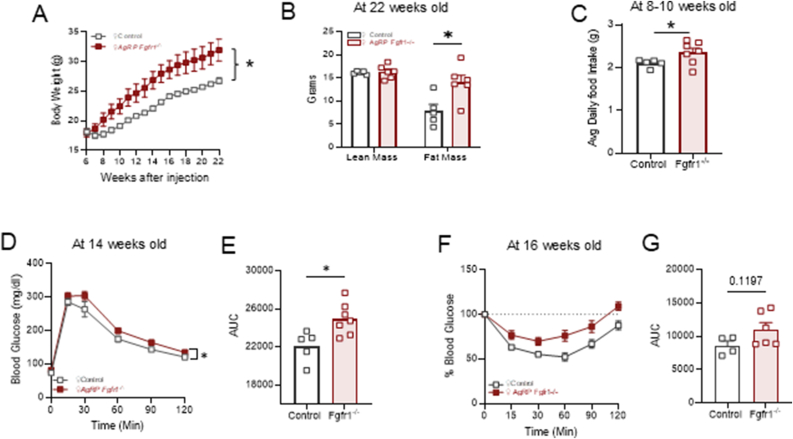
High-fat diet feeding induces weight gain, adiposity, and glucose intolerance following CRISPR/Cas9-mediated deletion of FGFR1 in AgRP neurons in female mice. A) Body weight curve after injection (control n = 6, AgRP FGFR1−/− n = 7). Two-way ANOVA (F (10, 160) = 133, P < 0.0001). B) Body composition analysis was performed at the age of 22 weeks old (n = 6 per group) unpaired students t tests two tailed (lean mass-t = 0.2697, df = 5.677, p = 0.796911; fat mass-t = 2.965, df = 8.964, p = 0.031520). C) Daily food intake was measured at the age of 8–10 weeks old (control n = 6, AgRP FGFR1−/− n = 7) Welch's two tailed t test, t = 2.338, df = 7.896, p = 0.048). D, E) Glucose tolerance test and AUC were performed at the age of 14 weeks old (control n = 6, AgRP FGFR1−/− n = 7) Two-way ANOVA F (10, 50) = 2.302, P = 0.0259; AUC Welch's two tailed t test t = 2.942, df = 8.941, p = 0.0165). F. G) Insulin tolerance test and AUC were performed at the age of 16 weeks old (control n = 6, AgRP FGFR11−/− n = 7) Two-way ANOVA F (1, 48) = 11.93, P = 0.0012; AUC Welch's two tailed t test t t = 1.742, df = 8 P = 0.1197). Data are presented as mean ± s.e.m. ∗P < 0.05.

Next, we subjected mice to a high-fat diet (HFD) feeding to determine if the DIO condition induces the metabolic role of FGFR1 in AgRP neurons. Male mice with deletion of FGFR1 in AgRP neurons showed increased weight gain (Figure 2A,B) and adiposity (Fig. 2C), and interestingly, these mice also showed increased lean mass, which may be a secondary byproduct of coupling overconsumption of HFD food rich in calories (Fig. 2D) with increased body mass and the need for ambulatory/locomotor function. Notably, during HFD feeding, mice lacking FGFR1 in AgRP neurons showed increased food intake compared to controls (Fig. 2D). Furthermore, deletion of FGFR1 in AgRP neurons led to worsened glucose tolerance (Figure 2E,F) and lower insulin sensitivity (Figure 2G,H), which may be accompanied by increased hepatic lipid accumulation (Figure 2I,J, and K) and expression of glucoregulatory enzymes PEPCK and G6Pase (Fig. 2L). The findings in male mice are consistent with the observed female phenotype (Supplementary Fig. 2) and align with prior reports demonstrating that pharmacological inhibition of FGFR1 in the hypothalamus increases food intake and body weight [6].

### 3.3 Deletion of FGFR1 in AgRP neurons leads to decreased energy expenditure

**Figure 3:**
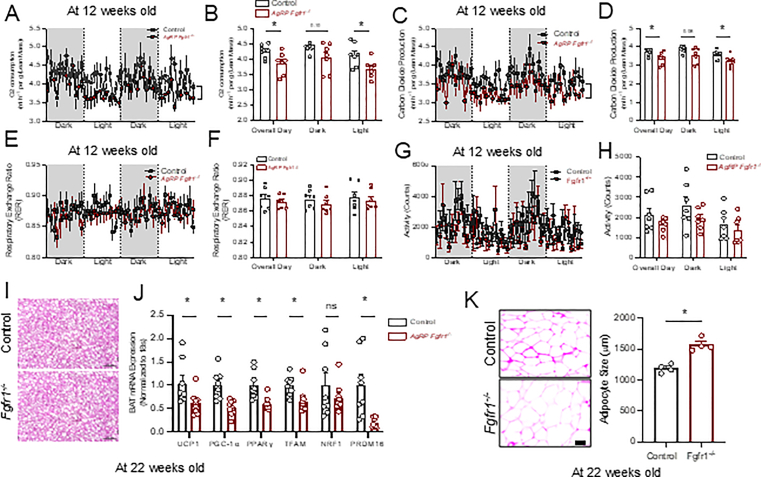
**Deletion of FGFR1 in AgRP neurons leads to decreased energy expenditure**. **A, B)** Normalized oxygen consumption (n = 7 per group) Two-way ANOVA F (1, 12) = 5.729, P = 0.0339). **C, D)** Normalized carbon dioxide production (n = 7 per group) Two-way ANOVA F (1, 12) = 5.419, P = 0.0382). **E, F)** Respiratory exchange ratio (n = 7 per group) Two-way ANOVA F (1, 12) = 0.5772, P = 0.4621). **G, H)** Locomotion/Activity counts (n = 7 per group) Two-way ANOVA F (1, 12) = 2.073, P = 0.1755). A-H was measured at the age of 12 weeks old**. I)** H&E of Brown Adipose Tissue. **J)** mRNA expression of thermogenic genes from BAT (n = 8 per group). **K)** WAT (Epididymal fat) H&E stains and quantification of WAT H&E adipocyte size (n = 4 per group) Welch's two tailed t test (t = 6.223, df = 5.187, P = 0.0014). I–K was measured at the age of 22 weeks old. Data are presented as mean ± SEM ∗*P* < 0.05, ∗∗*P* < 0.01.

Moreover, during HFD feeding, mice lacking FGFR1 in AgRP neurons showed reduced oxygen consumption (Figure 3A,B) and carbon dioxide production (Figure 3C,D), apart from changes in RER (Figure 3E,F) or locomotor activity (Figure 3G,H). In line with this finding, despite the absence of any observable morphological changes in brown adipose tissue resulting from AgRP neuron-specific FGFR1 deletion. (Fig. 3I), we observed a serial reduction of thermogenic gene expressions in BAT (*UCP1, PGC1-α*, *PPARγ, TFAM, and PRDM16),* which may be responsible for the reduced oxygen consumption in these mice lacking FGFR1 in AgRP neurons (Fig. 3J). As a result, the adipocyte size of epididymal fat in AgRP neuron FGFR1-deficient mice is significantly increased compared with that of the control (Fig. 3K). Together, these results demonstrate the metabolic importance of FGFR1 in AgRP neurons to mediate energy homeostasis during HFD feeding/obesogenic conditions.

### 3.4 Deletion of FGFR1 prevents α-klotho-mediated suppression of AgRP neuron activity

**Figure 4:**
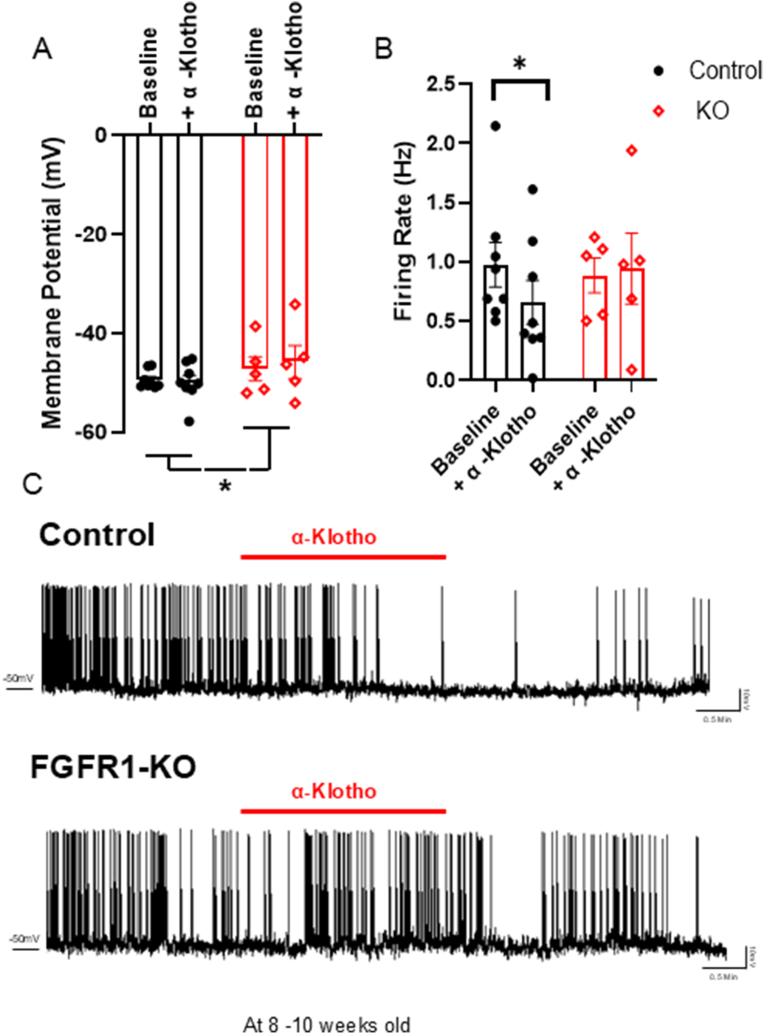
**Deletion of FGFR1 prevents α-Klotho-mediated suppression of AgRP neuron activity ex vivo**. **A)** Calculated membrane potential (mV), **B)** firing rate (Hz) of AgRP neurons following α-Klotho administration (3.65 mM) or saline (control n = 8, AgRP FGFR1−/− n = 5). Control two tailed paired t test, t = 2.473, df = 7, P = 0.0427), FGFR1-KO two tailed paired t test t = 0.1421, df = 4, P = 0.8939). **C)** Representative cell-attached recordings of AgRP neurons following α-Klotho administration (3.65 mM) or saline (control n = 8, AgRP FGFR1−/− n = 5). All data were collected from mice at the age of 8–10 weeks.

Ex vivo Patch-Clamp electrophysiological recording demonstrated that while the deletion of FGFR1 in AgRP neurons did not alter basic firing rates, the lack of FGFR1 in AgRP neurons depolarized resting membrane potential compared with control, regardless of the α-Klotho treatment (Figure 4A). Furthermore, α-Klotho suppressed AgRP neuron firing rates in control; however, α-Klotho stimulation did not affect the firing rates of AgRP neurons lacking FGFR1 (Figure 4B,C). Indicating α-Klotho activates FGFR1 in a cell-autonomous fashion (post-synaptic) to regulate AgRP neuron activity, as a previous report shows that recordings were performed in the presence of tetrodotoxin (TTX) (1.0 μmol/L), which blocks synaptic inputs [17].

### 3.5 Deletion of FGFR1 prevents α-klotho-mediated suppression of AgRP neuron activity in vivo

**Figure 5:**
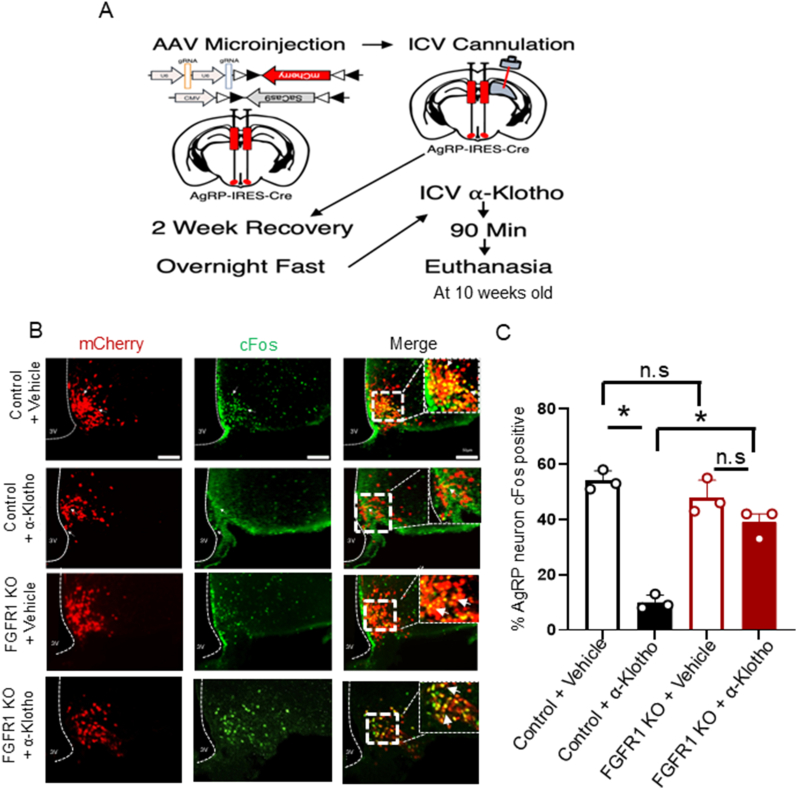
**Deletion of FGFR1 prevents α-Klotho-mediated suppression of AgRP neuron activity in vivo**. **A)** Schematic of experimental workflow and **B)** Representative immunofluorescent images of AgRP neuron (Red) colocalization with cFos (green) after an overnight fast and ICV administration of α-Klotho (3.65 mM) or saline. **C)** Quantification for the percentage of cFos colocalization in AgRP neurons. (n = 3 per group). Two-tailed unpaired t test t = 17.04, df = 3.670, P = 0.0001 for vehicle administrated vs α-Klotho administrated in control mice; two-tailed unpaired t test t = 1.919, df = 3.872, P = 0.1298 for vehicle administrated vs α-Klotho administrated in AgRP neurons FGFR1 deletion mice; two-tailed unpaired t test t = 1.441, df = 3.200, P = 0.2397 for vehicle administrated control mice vs vehicle administrated AgRP neurons FGFR1 deletion mice, and two tailed paired t test t = 8.614, df = 2.972, P = 0.0034) for α-Klotho administrated in control mice vs α-Klotho administrated in AgRP neurons FGFR1 deletion mice. All data were collected from mice at the age of 10 weeks old.

To determine whether FGFR1 in AgRP neurons was required for α-Klotho to suppress AgRP neuron activity in vivo, we administered (intracerebroventricular injection) α-Klotho into the lateral ventricle of AAV-injected mice following the recovery period of two weeks (Figure 5A). Consistent with ex vivo recordings and our previous findings that ICV administration of α-Klotho suppresses AgRP neuron activity [17]. After an overnight fast, administration of α-Klotho significantly reduced AgRP neuron activation (measured by cFos colocalization) in scramble gRNA-injected control mice compared to vehicle. This reduction was absent in mice lacking FGFR1 in AgRP neurons, which maintained elevated cFos levels. Baseline cFos did not differ between vehicle-injected control and knockout mice (Figure 5B,C). These results demonstrate that the inhibitory effect of α-Klotho on AgRP neuron activity in vivo requires FGFR1 signaling.

### 3.6 Loss of FGFR1 leads to dephosphorylation of FOXO1 in hypothalamic cells and AgRP mRNA upregulation in AgRP neurons

**Figure 6:**
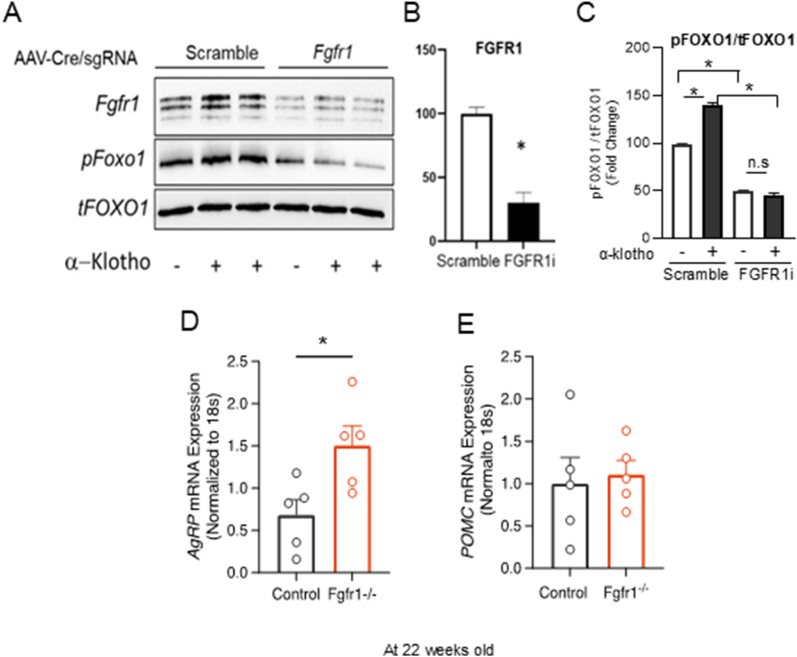
**Loss of FGFR1 leads to dephosphorylation of FOXO1 in hypothalamic cells and AgRP mRNA upregulation in AgRP neurons**. **A)** Immunoblot showing knockdown of FGFR1 by AAV-Cre/CRISPR/Cas9 blocks α-klotho induced phosphorylation of FOXO1. **B)** Quantification of FGFR1 expression (n = 3 per group, two-tailed unpaired t test t = 3.287, df = 4, P = 0.0303). **C)** Quantification of pFOXO1. (n = 2–3 per group, two-tailed unpaired t test t = 13.42, df = 2.439, P = 0.0024 for vehicle treated vs α-Klotho treated in control cells; two-tailed unpaired t test t = 27.46, df = 1.742, P = 0.0027 for vehicle treated in control cells vs vehicle treated in FGFR1 knockdown cells; two-tailed unpaired t test t = 30.7, df = 2.370, P = 0.0004 for α-Klotho treated in control cells vs α-Klotho treated in FGFR1 knockdown cells). **D)** AgRP mRNA expression from whole hypothalamus of AAV injected mice (n = 5 per group, two-tailed Welch's t test t = 2.761, df = 7.589, P = 0.0259). **E)** POMC mRNA expression from whole hypothalamus of AAV injected mice (n = 5 per group, two-tailed Welch's t test t = 0.3049, df = 6.118, P = 0.7706). Data are presented as mean ± s.e.m. ∗*P* < 0.05. D-E was collected at the age of 22 weeks old.

Next, to elucidate the molecular mechanisms of FGFR1 in AgRP neurons, we tested the ability of FGFR1 to activate the PI3K-FOXO1 pathway and subsequent phosphorylation of FOXO1 to reduce AgRP gene expression. Considering our previous data showing that α-Klotho reduces AgRP mRNA in hypothalamic GT1-7 cells, an effect that was blocked by the FGFR1 inhibitor PD 173074 or the PI3K inhibitor wortmannin [17], we questioned whether FGFR1 was required for these effects. Using CRISPR-Cas9-mediated knockdown of FGFR1 in hypothalamic GT1-7 cells, we found that knockdown of FGFR1 significantly reduced both the baseline phosphorylation of FOXO1 and its phosphorylation in response to α-Klotho (Figure 6A,B, C), providing evidence of an FGFR1-FOXO1 signaling axis in GT1-7 hypothalamic cells. Furthermore, we also observed upregulated AgRP mRNA expression (Fig. 6D) in the hypothalamus of mice lacking FGFR1 in AgRP neurons, while not influencing POMC mRNA expression (Fig. 6E). Together, this provides further evidence of the ability of FGFR1 to act in AgRP neurons to regulate both the electrical activity and the expression of AgRP mRNA, which is also highly associated with increased food intake, weight gain, and glucose level [34,35].

## 4 Discussion

This study elucidates a critical role for FGFR1 signaling within AgRP neurons in the central regulation of energy homeostasis. We demonstrate that FGFR1 acts as a key molecular on AgRP neuron activity, and its function is essential for mediating the anti-obesity effects of specific ligands, most notably α-Klotho. Our central finding is that the conditional deletion of FGFR1 specifically in mature AgRP neurons leads to a pronounced hyperphagic phenotype, characterized by increased weight gain, elevated adiposity, and impaired glucose tolerance. These metabolic deficits were significantly induced under the physiological challenge of a high-fat diet (HFD). This loss-of-function approach firmly positions FGFR1 in AgRP neurons as a potent regulator of metabolic physiology.

This finding helps reconcile previously conflicting reports on the role of central FGFR1 in metabolism [16,36]. While Liu et al. (2018) demonstrated that central FGFR1 inhibition via gut-derived bile acids improves glycemic control [36], Sun et al. (2023) conversely found that pharmacological activation of FGFR1 with FGF4 can reverse diabetes in rodent models [16]. The disparity in earlier studies likely stemmed from differences in animal models, the developmental timing of gene manipulation (e.g., constitutive vs. conditional knockout), and the specific neuronal populations targeted. Our approach, targeting FGFR1 deletion to a defined and metabolically critical neuronal subset in adulthood, avoids potential developmental compensation and precisely identifies AgRP neurons as a key site of action for FGFR1's metabolic effects. Although FGFR1 is abundant in the nervous system and was previously documented in glial cells [37], recent evidence shows it is expressed in nearly 90% of hypothalamic neurons [16]. The study crucially demonstrated that deleting FGFR1 in neurons, but not in astrocytes, impairs body weight and glucose regulation in mice, establishing neuronal FGFR1 in the medio-basal hypothalamus (MBH) as essential for metabolic control [16]. Aligning with this cell-type-specific paradigm, our data identify AgRP neurons as a major site mediating the metabolic functions of FGFR1.

Mechanistically, our research revealed that FGFR1 signaling governs AgRP neuron function through dual temporal mechanisms, modulating both acute neuronal activity and chronic transcriptomic states. It increases their firing rates and, by reducing FOXO1 phosphorylation, promotes AgRP expression, demonstrating that the α-Klotho–FGFR1–FOXO1 axis dually regulates neuronal excitability (short-term) and gene expression (long-term). The mechanism by which α-Klotho suppresses AgRP neuron activity likely involves PI3K signaling and subsequent hyperpolarization, a mechanism absent in neurons lacking FGFR1. AgRP neurons are hyperpolarized via the opening of ATP-sensitive potassium (KATP) channels in response to leptin and insulin through PI3K signaling [38]. Our previous finding that the PI3K inhibitor wortmannin blunts the therapeutic effects of α-Klotho [17] supports this as the underlying mechanism. This aligns with findings that the mechanism of FGF4 involves altering the excitability of medio-basal hypothalamic (MBH) neurons through KATP channels and the ABCC8 subunit [16]. Furthermore, CRISPR-Cas9-mediated deletion of the KATP channel subunit Kir6.2 (KCNJ11) in AgRP neurons is required for leptin action [39], suggesting that KATP channel function is critical for these metabolic effects, regardless of the specific subunit. Additionally, a decreased firing rate without hyperpolarization that was observed in the current study suggests α-Klotho modulates channels controlling excitability, rather than resting potential. Such changes can arise from enhanced potassium currents that accelerate repolarization [40] or altered sodium channel kinetics that elevate firing threshold [41] or reduction of subthreshold depolarizing conductances [42]. This points to a gain-control mechanism that fine-tunes AgRP neuron output without shifting baseline membrane excitability.

On a longer-term transcriptional scale, the loss of FGFR1 signaling significantly increased hypothalamic AgRP mRNA. This indicates that constitutive FGFR1 signaling normally suppresses AgRP gene expression. In FGFR1-deficient AgRP neurons, phosphorylation of FOXO1—a transcriptional repressor of AgRP—was reduced under both basal and α-Klotho-stimulated conditions in hypothalamic cells. This decrease likely results from attenuated basal PI3K-AKT signaling [17], leading to less active (more dephosphorylated) FOXO1. Consequently, the increased transcriptional activity of FOXO1 enhances orexigenic tone, including the promotion of AgRP gene expression (Fig. 6D). Together, the absence of FGFR1 signaling enhances both AgRP transcription and neuronal excitability, synergistically promoting hyperphagia and energy conservation, ultimately driving the observed metabolic phenotype. Future studies are warranted to investigate whether constitutive FGFR1 signaling exerts a tonic inhibitory influence on AgRP neuron activity in DIO mice, a finding essential for elucidating its mechanistic role in obesity.

Beyond α-Klotho, FGFR1 is a receptor tyrosine kinase for multiple FGF ligands, including FGF21 and others known to have central metabolic effects [[5], [6], [7], [8],[12], [13], [14], [15],^43^]. Our work provides a plausible cellular and molecular mechanism for the actions of these systemic factors. We speculate that AgRP neurons represent a central hub where various metabolic FGFs, potentially through FGFR1, converge to regulate energy balance. A key future direction will be to determine the specific contributions of other FGF ligands versus α-Klotho in activating this receptor under different physiological conditions (e.g., fasting vs. feeding, lean vs. obese states). For instance, the finding that long-term diabetic remission induced by FGF1 occurs in hyperglycemic but not normoglycemic animals [5] suggests that its effects are state-dependent, gaining importance in metabolically compromised states. This effect is hypothesized to involve a restorative impact on neurocircuits governing energy balance to correct dysfunctional signaling in metabolic disease. Notably, since models such as leptin-deficient (ob/ob) or leptin receptor-deficient (db/db, ZDF rats) mice were used in that study [5], this raises the possibility that FGF/FGFR1 signaling in AgRP neurons subserves a critical metabolic function analogous to leptin signaling, potentially compensating for defective leptin receptor (LepR) signaling to maintain energy and glucose homeostasis. Several facts support this plausibility: (i) the ARC is primarily composed of GABAergic neurons that regulate energy homeostasis [44] (ii) AgRP neurons are GABAergic, and their GABA release is required for proper energy balance [45]; (iii) α-Klotho [17], FGF19 [7], and FGF1 [37] all inhibit AgRP neuron activity; and (iv) the ability of ICV FGF19 to elicit “leptin-like” effects is blunted in obese Ay mice, where ubiquitous agouti expression blocks MC4R signaling [7]. This collective evidence strongly implicates the metabolic role of FGF1/FGFR1 in AgRP neurons in mediating these phenotypes, though precise mechanisms require further elucidation. While this study did not aim to delineate the contribution of each specific FGFR1 ligand (e.g., FGF1, FGF19, FGF21, FGF4), our findings demonstrate that selective deletion of FGFR1 in AgRP neurons of adult mice alters energy homeostasis, promoting an obesogenic state, especially under HFD, highlighting its essential role. We also provide evidence for the FGFR1 signaling in AgRP neurons that acts similarly to leptin and insulin, regulating both the electrical properties and gene expression of the AgRP neuropeptide.

While our findings demonstrate that AgRP neuron-specific FGFR1 deletion promotes diabetic parameters, it remains unclear whether these effects are driven directly by the loss of FGFR1 signaling in AgRP neurons or are secondary to the resulting increase in adiposity. Distinguishing neuron-autonomous mechanisms from adiposity-dependent effects will be a critical objective for future studies.

## 5 Conclusion

Our results define a crucial hypothalamic pathway wherein FGFR1 signaling in AgRP neurons tonically suppresses activity and gene expression to maintain metabolic equilibrium. The necessity of this receptor for α-Klotho's efficacy highlights its potential therapeutic relevance. Targeting the FGFR1 signaling pathway specifically within the AgRP neural circuit could represent a novel and highly precise strategy for treating obesity and type 2 diabetes by restoring proper signaling in individuals with AgRP neuron dysfunction or resistance to metabolic hormones.

## CRediT authorship contribution statement

**Daniel Shookster:** Validation, Investigation, Formal analysis, Data curation. **Shea O'Connell:** Writing – review & editing, Validation, Methodology, Investigation. **Patel Darshan:** Writing – review & editing, Validation, Methodology, Investigation, Data curation. **Taylor Landry:** Methodology, Investigation, Conceptualization. **Wyatt Bunner:** Software, Resources, Methodology. **Zhiying Jiang:** Visualization, Validation, Methodology, Formal analysis, Data curation. **Qingchun Tong:** Validation, Supervision, Resources, Methodology, Investigation, Funding acquisition. **Hu Huang:** Writing – review & editing, Writing – original draft, Supervision, Project administration, Investigation, Funding acquisition, Conceptualization.

## Guarantor statement

Dr. Hu Huang is the guarantor of this work and, as such, has full access to all the data in the study and takes responsibility for its integrity and the accuracy of the analysis.

## Ethics declaration

The authors declare that the research was conducted in the absence of any commercial or financial relationships that could be construed as a potential conflict of interest.

## Funding

The funding for this project was provided by 10.13039/100000002NIH R15DK121215, DK139539 to Hu Huang. Additional funding was provided by NIH R01DK135212, DK131446, DK136284, DK109934, and 10.13039/100000005DOD HT94252310156 to Qingchun Tong.

## Declaration of competing interest

The authors declare that the research was conducted in the absence of any commercial or financial relationships that could be construed as a potential conflict of interest.

## Data Availability

Data will be made available on request.
