# Supplementary material for: Selective deletion of FGFR1 in AgRP neurons impairs energy homeostasis under high-fat diet in mice
Source: Mol Metab. 2026 Feb 10;105:102332. doi: 10.1016/j.molmet.2026.102332 (PMC12936525; doi:10.1016/j.molmet.2026.102332)
Supplement: Multimedia component 1 [file mmc1.docx]

**Supplemental Table 1**: Primer Sequences used in Quantitative real-time PCR
